# Burnout Assessment Tool (BAT)—Development, Validity, and Reliability

**DOI:** 10.3390/ijerph17249495

**Published:** 2020-12-18

**Authors:** Wilmar B. Schaufeli, Steffie Desart, Hans De Witte

**Affiliations:** 1 Research Group Work, Organizational and Personnel Psychology (WOPP), O2L, KU Leuven, 3000 Leuven, Belgium; steffie.desart@idewe.be (S.D.); hans.dewitte@kuleuven.be (H.D.W.); 2 Department of Social and Organizational Psychology, Utrecht University, 3584 CS Utrecht, The Netherlands; 3 Optentia Research Focus Area, North-West University, Vanderbijlpark 1900, South Africa

**Keywords:** burnout, conceptualization, scale development, validation, Burnout Assessment Tool (BAT)

## Abstract

This paper introduces a new definition for burnout and investigates the psychometric properties of the Burnout Assessment Tool (BAT). In a prior qualitative study, 49 practitioners were interviewed about their conceptualization of burnout (part 1). Using a dialectical approach, four core dimensions—exhaustion, mental distance, and impaired emotional and cognitive impairment—and three secondary dimensions—depressed mood, psychological distress, and psychosomatic complaints—emerged, which constitute the basis of the BAT. In the second study, the psychometric characteristics of the BAT were investigated in a representative sample of 1500 Flemish employees, focusing on factorial validity, reliability, and construct validity, respectively. Results demonstrate the assumed four-factor structure for the core dimensions, which is best represented by one general burnout factor. Contrary to expectations, instead of a three-factor structure, a two-factor structure was found for the secondary dimensions. Furthermore, the BAT and its subscales show adequate reliability. Convergent validity and discriminant validity with other burnout measures—including the MBI and OLBI—was demonstrated, as well as discriminant validity with other well-being constructs, such as work engagement and workaholism.

## 1. Introduction

From the outset, the assessment of burnout has been debated. Most research has used—what has become the golden standard of burnout—the Maslach Burnout Inventory (MBI) [1]. It has been estimated that the MBI is used in 88% of all publications on burnout [2]. The MBI contains three factors, originally labelled as emotional exhaustion, depersonalization, and reduced personal accomplishment. This version was later dubbed MBI—Human Service Survey (MBI-HSS) [3] and adapted for educators (MBI—Educator Survey; MBI-ES) [3] and medical personnel (MBI—HSS-MP) [4]. Because of its original definition and wording (i.e., most items refer to “recipients”, “patients”, or “students”), these versions of the MBI are specific for use within human services or educational and medical settings. Later, the definition of burnout was broadened to include not only those “who do people work of some kind” ([1], p. 99) but employees in *every* kind of job. To assess this broadened burnout concept a general version was developed, the MBI-General Survey (MBI-GS) [5]. The three components of the MBI-GS are equivalent to those of the MBI-HSS/ES: (1) *exhaustion*, the depletion of one’s mental resources at work; (2) *cynicism*, a distant attitude toward the job; and (3) *reduced professional efficacy,* a lack of achievement and productivity at work. Essentially, the MBI-GS assesses the same three dimensions as the original measure by using more general worded items that refer to one’s job and not specifically focus on recipients. 

Despite its popularity, we have identified three flaws with the MBI. First, there are problems with the conceptualization of burnout. Meanwhile, research has consistently linked burnout to cognitive malfunctioning and deficits as well (for an overview, see [6]). Moreover, it was documented that particular distress symptoms, such as irritability, sleeping problems, and tension headaches, seem to occur in employees who suffer from burnout. These so-called neurasthenic complaints even led some authors to consider burnout as a work-related type of neurasthenia [7]. In addition, there is an on-going debate about the relationship between burnout and depression, whereby some have argued that burnout is merely an atypical depressive disorder [8], whereas others maintain that burnout and depression do not overlap and are “two different robust constructs” ([9], p. 1). Although most authors might not agree (e.g., [10]), it is nevertheless clear that depressive and burnout symptoms often co-occur and develop in tandem [11]. We also question whether if reduced professional efficacy is a constituent part of burnout [12]. 

Second, the MBI suffers from technical, psychometric shortcomings. To start, the extreme formulation of some of its items (e.g., “I feel I treat some recipients as if they were impersonal objects”) may lead to low reliabilities, especially for the subscales assessing cynicism and reduced professional efficacy (for a meta-analysis, see [13]). This meta-analysis concludes that: “Of the three MBI subscales, Personal Accomplishment and Depersonalization mean alpha estimates were well below recommended levels for high-stakes decisions, such as the diagnosis of burnout syndrome” (p. 231). Furthermore, it was shown that reversing the positively worded professional efficacy items in order to indicate a *lack* of professional efficacy, introduces an artefact. Accordingly, correlations of the reversed positively worded efficacy scale are much lower than when a negatively worded scale is used [14]. Moreover, the factorial validity of the MBI is questioned. For instance, de Beer and Bianchi [15] showed that the subscales of emotional exhaustion and cynicism seem to represent a common factor, while a separate, second factor represents the professional efficacy scale. This is in line with the doubts that have been raised about the role of efficacy or accomplishment in burnout. 

Third, the practical applicability of the MBI for individual burnout assessment is rather poor. A key issue when it comes to norms as well as to predictive validity, is the fact that the MBI does not produce a single burnout score that can be dichotomized in order to distinguish between burned-out and non-burned-out cases. The MBI test manual explicitly states: “In general, each respondent’s scale scores should be calculated and interpreted separately. Note that responses to MBI items should not be combined to form a single “burnout” score” ([4], p. 44). Particular in the European context, identification of burnout cases is essential because European welfare states require a formal diagnosis as an “entrance ticket” for social and medical services, such as sickness and work incapacitation pensions, and prevention and treatment programs for burnout (e.g., Sweden and The Netherlands). In order to help practitioners with diagnosing burnout, an assessment tool—in the form of a self-report questionnaire—is of great importance. The MBI cannot play this role because it was developed as a multi-dimensional research instrument and *not* as an individual assessment tool. Although recently the WHO [16] included burnout in the newest version of the International Classification of Diseases (ICD-11), it was not included as a disease that should be diagnosed accordingly but as an “occupational phenomenon”. Hence the stance of the WHO is ambivalent on the one hand by including burnout in their list of diseases, whilst on the other hand by denying that it is one. To add to the confusion, the burnout definition of Maslach and colleagues [1] is adopted, thereby implicitly stating the MBI should be used to assess that occupational phenomenon. Hence, this does not solve the problem of assessing burnout as an occupational disease [17].

Although a number of alternative burnout questionnaires have been proposed, no instrument meets all concerns mentioned above. For instance, some one-dimensional questionnaires reduce burnout to mere exhaustion, thereby ignoring its multi-faceted nature (e.g., Burnout Measure (BM) [18]); Shirom Melamed Burnout Measure [SMBM] [19]; and the Copenhagen Burnout Inventory (CBI) [20]). Other multi-dimensional questionnaires use a similar conceptualization and the same subscales as the MBI, except that the wording of the items differs (e.g., Bergen Burnout Inventory (BBI) [21]); Granada Burnout Questionnaire (GBQ) [22]), probably in an attempt to circumvent the copyright protection of the MBI. Finally, the Oldenburg Burnout Inventory (OLBI) [23] assesses two dimensions of burnout—exhaustion and disengagement—but uses negatively as well as positively worded items. This procedure is considered problematic as positively framed “burnout” items are likely to tap its opposite—work engagement [24]. 

Thus, in order to overcome the flaws of the MBI related to its conceptualization, psychometric shortcomings, and practical applicability, we developed a novel burnout instrument that is suited for group- and individual-based assessment of burnout. Hence, our research has two aims:

1. Formulate an alternative conceptualization of burnout, which is comprehensive in nature and includes all relevant elements that are associated with burnout as conceived by practitioners.
2. Develop—based on this new conceptualization—a novel questionnaire that is psychometrically sound and practically useful for the assessment of burnout, dubbed Burnout Assessment Tool (BAT).

Each aim is pursued in a separate part that is qualitative and quantitative in nature, respectively.

## 2. Part 1: Conceptualization and Constructing of the BAT

In the first phase of part 1, burnout is redefined using a dialectic method that combines a deductive and inductive approach, allowing us to integrate insights from both practice and theory. In the second phase, questionnaire items are formulated after carefully scrutinizing items from existing burnout instruments. 

### 2.1. Phase 1. Redefining Burnout 

Reconceptualising burnout is an essential part of the construction of the BAT. Hence, the purpose of this first phase is to establish a conceptual framework. To reach this goal, in-depth, semi-structured interviews were conducted with practitioners, who deal with burnout on a daily basis. A long-list of burnout symptoms emerged that were clustered and interpreted using the conceptual framework of burnout formulated by Schaufeli and Taris [12]. Following the grand old man of psychological fatigue research, Edward Thorndike [25], who argued that the basic tenet of fatigue is “the intolerance of any effort”, Schaufeli and Taris [12] theorized that burnout is the combination of the inability and the unwillingness to no longer spend the necessary effort at work for proper task completion. In their view, “inability” manifests itself in lack of energy and “unwillingness” in increased resistance, reduced commitment, lack of interest, and disengagement. In fact, inability and unwillingness constitute two inseparable components, which lie at the heart of the burnout phenomenon, representing its energetic and motivational dimension, respectively. Both are inherently linked and can be seen as both sides of the same (burnout) coin.

#### 2.1.1. Method

Three types of professionals were interviewed: (1) General practitioners, to whom patients turn with burnout complaints; they are familiar with the patient and usually assess burnout (*n* = 19); (2) psychologists, who council or treat those with severe burnout complaints (*n* = 17); (3) occupational physicians, who decide whether or not workers with burnout complaints are fit for work (*n* = 13). By using a mixed group of 49 practitioners, who are involved at the beginning, middle, and end of the burnout process, a comprehensive and interdisciplinary understanding of the phenomenon is achieved. 

*Procedure*. The in-depth, face-to-face, semi-structured interviews lasted about one hour and were held in the spring of 2016. Interviewees were asked to describe a patient with prototypical burnout symptoms and to focus on specific symptoms, causes, and the way burnout unfolds across time. They were also invited to describe burnout in their own words, and to prioritize the burnout symptoms they mentioned in terms of their relevance for diagnosing burnout. 

*Data analysis*. In order to group the burnout symptoms, the in-depth interviews were content analysed using the Computer Assisted Qualitative Data Analysis program Nvivo. This program clustered specific symptoms that emerged in the interviews into a number of distinct groups [26]. First, all symptoms mentioned were given a single code and these codes were summarized on a coding sheet. For instance, the symptom “When encountering a complex situation that requires attention, difficulties arise in dealing with it” was coded as “difficulties with complex situations” Next, broader categories were generated. For instance, codes like “difficulties with complex situations” were placed under the category “attention and concentration deficits”. The grouping into new categories stopped when no new categories emerged from the coding sheets with symptoms (thematic saturation; see [27]). Finally, dimensions were constituted by grouping similar categories. Each dimension was labelled in such a way that it captured the underlying categories and codes best [28]. For instance, the category “attention and concentration deficits” was part of the dimension “cognitive impairment”. 

#### 2.1.2. Results

In total, 260 codes or symptoms were collected on the coding sheet, which were clustered into 19 categories. Eventually, seven dimensions emerged: (1) Exhaustion, (2) mental distance, (3) emotional impairment, (4) cognitive impairment, (5) depressed mood, (6) psychological distress, and (7) psychosomatic complaints. 

These seven dimensions were further clustered into primary dimensions and secondary dimensions based on the theoretical reasoning of Schaufeli and Taris [12]. The primary dimensions can be seen as a form of either “inability”, captured by exhaustion, impaired emotional, and cognitive control, or “unwillingness”, captured by mental distancing. Exhaustion or extreme tiredness is the most obvious symptom that was identified unanimously by all practitioners and refers to a severe and serious loss of energy, both physical as well as mental. All practitioners considered exhaustion a necessary but not sufficient condition for burnout. In addition, emotional and cognitive impairment were identified as constituting elements or core dimensions of burnout. The former refers to the reduced functional capacity to adequately regulate one’s emotional processes such as anger or sadness, whereas the latter refers to the reduced functional capacity to adequately regulate one’s cognitive processes, such as memory or attention. These functional capacities are impaired because of a lack of energy; in that sense, lacking energy is paramount. The final constituting element of burnout is mental distance, referring to mental withdrawal and psychological detachment from the job. This can be seen as a coping strategy to deal with feelings of exhaustion. However, this coping attempt is ineffective because it increases stress at work—for instance, because it might cause conflicts with colleagues or clients—and hence exacerbates the employee’s feelings of exhaustion. 

The four core dimensions are accompanied by three secondary dimensions: (1) Depressed mood, a common reaction to disappointment or loss that should be distinguished from mood disorder or a major depression, which is a psychiatric disorder; (2) psychological distress, or unpleasant feelings that are associated with high arousal and have a negative impact on the level of functioning and interfere with daily activities; and (3) psychosomatic complaints; physical symptoms that are thought to be caused, or exacerbated, by psychological factors. These three symptoms are considered to be secondary to the syndrome of burnout because they are atypical and may also appear in other physical and mental disorders, such as hyperthyroidism, cancer, mood disorder, or anxiety disorder. Furthermore, given the conceptual framework of Schaufeli and Taris [12], these dimensions neither reflect the inability nor the unwillingness to spend necessary effort at work. Nevertheless, these secondary symptoms are important because they are often the reason why employees seek help or assistance. 

Based on the considerations above, burnout is defined as: “a work-related state of exhaustion that occurs among employees, which is characterized by extreme tiredness, reduced ability to regulate cognitive and emotional processes, and mental distancing. These four core dimensions of burnout are accompanied by depressed mood as well as by non-specific psychological and psychosomatic complaints”. 

Please note that burnout is defined as a work-related mental state, whereby work is *not* restricted to paid employment but viewed from a broader, psychological perspective. Psychologically speaking “work” refers to those structured, goal-directed activities that are mandatory in nature and requires exerting oneself against the environment ([29], p. 57). Following this line of reasoning, the activities of athletes, volunteers, and students can be seen as “work”, and hence, they may also suffer from burnout. Recently, it has been claimed that parents can suffer from burnout as well [30]. This so-called parental burnout is characterized by an overwhelming exhaustion related to one’s parental role, an emotional distancing from one’s children, and a sense of parental ineffectiveness. 

### 2.2. Phase 2. Item Formulation

#### 2.2.1. Method

*Procedure*. Before formulating the items for each dimensions of the BAT, we first examined the item content of existing burnout questionnaires. A literature review was carried out using the search engines Limo, Web of Science, and Google Scholar, resulting in 20 questionnaires using the terms “burnout”, “burn-out”, and “burn out” combined with other terms like “questionnaire”, “survey”, “assessment”, “measurement”, “test”, “inventory”, “checklist”, and “measure”. Only questionnaires in English, Dutch, French, German, or Spanish were retained. Four types of questionnaire were distinguished: (1) Questionnaires with known validity and reliability (k = 9); (2) questionnaires with unknown validity and reliability (k = 3); (3) questionnaires that assess a specific forms of burnout, such as the Physicians Burnout Questionnaire (PhBQ) [31] (k = 8). Only (1) and (2) were considered next because the wording of (3) referred to specific professions. In addition, the four-Dimensional Symptom Questionnaire (4-DSQ) was considered, which has been developed for use in the general population [32]. Besides, the use of the 4-DSQ is recommended in the officially sanctioned guidelines for the diagnoses of stress-related adjustment disorders for general practitioners and occupational physicians [33]. Furthermore, its validity as a screening tool was demonstrated in an occupational health context [34]. The 4-DSQ does not measure burnout per se, but focuses on mild symptoms of depression, anxiety, somatization, and distress in primary healthcare patients. 

All 12 questionnaires from groups 1 and 2 (including the 4DSQ) were further scrutinized in terms of scoring, subscales, conceptual model, and wording of the items (see Appendix A Table A1). After careful consideration, we decided not to incorporate items that reflect a depressed mood in the BAT, because other, short, well-validated depression questionnaires are available that can be used in an occupational health context, such as the depression subscale of the 4-DSQ [32].

#### 2.2.2. Results

A complete list of 357 items and 66 dimensions was drafted. On average, each questionnaire contained three to four dimensions. Exhaustion was the only dimension included in *all* questionnaires. Three questionnaires even restricted burnout to mere exhaustion (i.e., BM, SMBM, CBI) while one questionnaire only measured exhaustion and secondary symptoms (i.e., BO-NKS) [35], and one questionnaire only focused on the secondary symptoms (4-DSQ). The remaining eight questionnaires defined burnout as a multidimensional construct and included at least two scales that measured exhaustion and mental distance. All questionnaires used a Likert-scale that assesses the frequency of the burnout symptoms, but the number of scale points ranged from four to seven. Furthermore, most scales used negatively worded items (76%), whereas only five scales (7%) used items that are positively worded and 11 scales (17%) used negatively as well as positively worded items. 

Hence, it can be concluded that: (1) There is general consensus that exhaustion is the most essential dimension of burnout; (2) all multi-dimensional questionnaires include both exhaustion as well as mental distance, which agrees with the conceptual framework of Schaufeli and Taris [12]; (3) positively framed items are the exception rather than the rule; and (4) a Likert-scale type of frequency response format is used with four to seven anchors. 

Based on these conclusions, each author formulated five items for each of the six dimensions; hence, in total, 90 items were formulated, 15 per dimension. These items were used as the input for a consensus discussion among the authors that led to a 33-item version of the BAT. This includes four core dimensions, further referred to as BAT-C (see Appendix B Table A2), and two types of secondary dimensions, further referred to as the BAT-S (see Appendix B Table A3). The BAT does not include a subscale for depressed mood, but we recommend using the 6-item depression subscale from the 4-DSQ [32]. 

## 3. Part 2: Validity and Reliability of the BAT

In the second part, we focussed on the psychometric qualities of the BAT. First, the factorial validity was assessed by using an exploratory (EFA) as well as confirmatory factor analysis (CFA). Second, the reliability was evaluated by assessing the internal consistency (Cronbach’s, α) of each subscale and the composite BAT-C and BAT-S scales. Third, the convergent validity (i.e., the degree to which it converges with other burnout instruments) and discriminant validity (i.e., the degree to which core aspects of burnout can be discriminated from each other) of the BAT was tested by using the multi-trait, multi-method (MTMM) framework of Campbell and Fiske [36]. More specifically, this framework was used to study: (1) The convergent and discriminant validity of the BAT-C vis-à-vis the MBI-GS and the OLBI; (2) the external discriminant validity of the BAT by investigating its relationships with other well-being constructs, such as work engagement, workaholism, and job boredom (e.g., [37]). Work engagement is described as “a positive, fulfilling, work related state of mind that is characterized by vigor, dedication, and absorption” ([38], p.74). Whilst some scholars argue that burnout and work engagement are each other’s opposites (e.g., [39]), others consider both as being correlated, but independent constructs (e.g., [38]). At any rate, burnout and work engagement are negatively related [40]. Workaholism is defined as the uncontrollable inner need to work extremely hard [41], and includes a behavioural (working excessively) as well as a cognitive (working compulsively) dimension. In the 1980s, workaholism was already identified as a possible cause for burnout [3]. A recent longitudinal study, spanning four years, confirmed that workaholism leads to burnout and not the other way around [42]. Most likely, workaholics often do not take the opportunity to recuperate from their efforts, leading to a progressive loss of energy [43]. In short, burnout and workaholism are distinct but positively related concepts. Job boredom is defined as an unpleasant state that is characterized by relatively low arousal and dissatisfaction that results from inadequate stimulation at work [44]. It is usually seen as the consequence of understimulation, while burnout is seen as a consequence of overstimulation. Although both are characterized by feeling worn-out, bored employees feel less negative and more active than employees who suffer from burnout [45] (Schaufeli & Salanova, 2014). Hence, a positive relationship is expected between burnout and job boredom. 

### 3.1. Method

#### 3.1.1. Participants

A sample of 1500 employees was collected with the aid of an online panel provider (iVOX) in the spring of 2017. The sample was representative for the Flemish working population based on age, gender, and economic sector (http://statbel.fgov.be). Age and gender were used as “hard quota” for which the stratification was perfect, while economic sector was considered a “soft quotum”, meaning that a slight deviation of 2% from the population was allowed. About 54% of the participants were male and the average age of the participants was 41 years old (SD = 12). In total, 21% worked in the primary or industrial sector, 47% in the service sector, 12% in the public sector, 8% in the education sector, and 12% in the healthcare sector. The project "Development and validation of a questionnaire to assess burnout” was approved by the Social and Societal Ethics Committee of KU Leuven on October 22nd 2015 (#G-2015-10-353).

#### 3.1.2. Measures

*Burnout* was measured with three questionnaires. As outlined above, the total BAT contains 33 items and consists of the BAT-C and BAT-S. The BAT-C assesses the four core dimensions—exhaustion, mental distance, impaired emotional, and cognitive control—and contains 23 items, while the BAT-S assesses the two secondary dimensions—psychological and psychosomatic complaints—and contains 10 items. Both are rated on a five-point Likert scale ranging from never (1) to always (5). 

The Utrecht Burn-Out Scale (UBOS-A)—the Dutch equivalent of the MBI-GS [46]—is a 15-item questionnaire that measures burnout using three dimensions—exhaustion, cynicism, and professional efficacy (α = 0.92, 0.87, and 0.84, respectively). Items are rated on a seven-point Likert scale from never (1) to daily (7). 

The OLBI [23] is a 16-item burnout instrument that includes two dimensions—exhaustion and disengagement (α = 0.79 and 0.90, respectively). Items are scored on a four-point Likert scale, ranging from strongly disagree (1) to strongly agree (4). 

*Work engagement* was measured using the ultra-short version of the Utrecht Work Engagement Scale (UWES) [47], which contains three items that refer to vigour, dedication, and absorption, (α = 0.84). Items are scored on a five-point Likert Scale, ranging from never (1) to always (5). 

*Workaholism* was assessed using the Dutch Work Addiction Scale (DUWAS) [48]. This 10-item questionnaire includes two dimensions: working excessively (α = 0.76) and working compulsively (α = 0.78). Items are rated on a seven-point Likert scale from never (1) to always (5). 

*Job boredom* was measured with the Dutch Boredom Scale (DUBS) [49] and contains six items (α = 0.85). This questionnaire is again scored on a five-point Likert Scale, ranging from never (1) to always (5). 

*Depressed mood* was assessed with the 6-item subscale for depression of the 4-DSQ [32] (α = 0.93), which is scored on a five-point Likert Scale, ranging from never (1) to always (5).

#### 3.1.3. Data Analysis

*Preliminary analysis.* First, the skewness and kurtosis of the score distributions of the BAT items were examined in both samples separately ([4] more detailed information about the score distribution of the items is available upon request from the first author). At first glance, the values of these distribution characteristics did not give cause for concern as they range from |0.17| to |1.16|. Because of the large sample size, it does not make sense to conduct a formal test for normality, since even very small and irrelevant deviations from normality will be statistically significant ([50]; pp. 183–186). In that case, the normality of the distribution can best be assessed by visual inspection of the score distributions. From this it could be concluded that the scores for exhaustion and cognitive impairment are approximately normally distributed. However, this was not always the case for the other two core symptoms; respondents were relatively less bothered by mental distance and emotional impairment. Furthermore, some items of the secondary burnout symptoms were normally distributed, while others were not. The statistical analyses used below are fairly robust for violations of the assumption of normality, though. In other words, it is unlikely that departures from normality influenced the results of our analysis.

*Factorial validity*. By means of cross-validation, the sample was randomly split in half. In the first subsample (*n* = 750)—the developmental sample—the structure of the BAT was examined by using an exploratory factor analysis (EFA); i.e., Principal Axis Factoring with oblimin rotation in SPSS 23. In the second subsample (*n* = 750)—the validation sample—the factor structure that emerged from the development sample was cross-validated by using Confirmatory Factor Analysis (CFA) with MLM maximum likelihood parameter estimation in Mplus 8.1.

Using the developmental sample, separate EFA’s were conducted for the core BAT items and for the secondary BAT items plus the depression subscale of the 4-DSQ, respectively. The factors were extracted using the principal axis factoring method, followed by an oblique rotation (direct oblimin with Kaiser normalization). The suitability of our data was evaluated with the Kaiser–Meyer–Olkin (KMO) measure of sampling adequacy and Bartlett’s test of sphericity. As a rule of thumb, 0.40 is considered as the minimal required loading of an item, while cross-loading items are defined as items loading 0.30 or higher on two or more alternative factors [50]. 

Using the validation sample, several models were tested by means of CFA. For the core symptoms of the BAT-C, three models were evaluated. The first model (Model 1) is a one-factor model in which all items load on one general burnout factor. The 4-factor correlated model (Model 2) assumes four distinct but correlated factors—exhaustion, mental distance, and impaired emotional and cognitive control. Since burnout is supposed to be a syndrome that consists of a set of related symptoms that refer to one underlying psychological condition, a second-order model was tested as well (Model 3). This hierarchical model assumes four distinct factors that are indicators of one general, underlying factor (i.e., the core of burnout). This higher-order factor is supposed to be the reason why the four factors are correlated [51]. 

For the secondary symptoms of the BAT-S and the depression subscale of the 4-DSQ, two models are tested. As with the BAT-C, a one-factor model (Model 4) and a correlated factor model (Model 5) were tested. Given that only two factors emerged from the EFA, a hierarchical model is not feasible because it would entail an underestimation of the direct effect of the second-order factor or the error variances [52]. Model 4 assumes that all items load on one general factor, while Model 5 assumed two distinct factors, psychological and psychosomatic complaints, and depressed mood. 

Next, three additional models were tested that combine the core and secondary dimensions. Model 6 is a correlated factor model and assumes that six distinct factors can be distinguished. Additionally, two hierarchical models were tested. Model 7 assumes that all six distinct factors are best captured by one general, second-order factor (i.e., burnout), while Model 8 assumes that the four core factors are best captured by a first general factor (i.e., the core of burnout), while the remaining two factors are best captured by a second general factor (i.e., secondary symptoms). This last model adheres to the conceptualization of burnout as outlined above, which makes a distinction between core and secondary dimensions of burnout. 

In order to evaluate goodness-of-fit four fit indices are used [53]: Chi-square (χ^2^), comparative fit index (CFI), Tucker-Lewis index (TLI), and the root mean square error of approximation (RMSEA). A model fits the data well when CFI and TLI exceed at least 0.90 but preferably 0.95, and RMSEA is less or equal than 0.06 [54]. 

*Reliability*. The reliability is evaluated in terms of internal consistency, as based on Cronbach’s alpha. 

**Figure 1:**
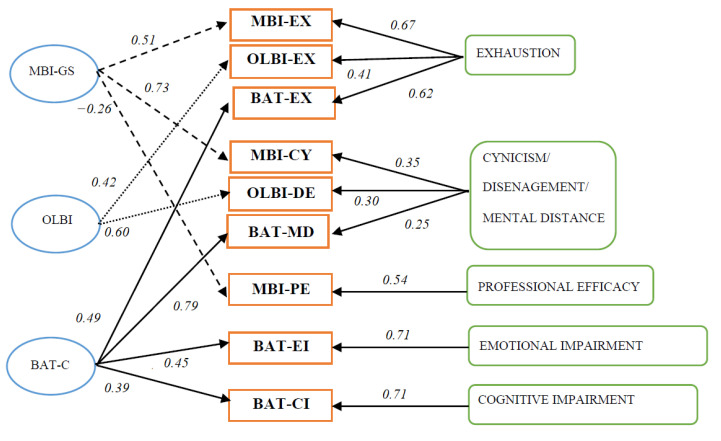
Multi-trait, multi-method framework for the Burnout Assessment Tool—Core (BAT-C), Maslach Burnout Inventory-General Survey (MBI-GS), and Oldenburg Burnout Inventory (OLBI) (correlated traits–correlated methods (CT-CM) model) with average pad coefficients.

*Construct validity*: *Convergent and discriminant validity with other burnout measures*. In order to establish convergent and discriminant validity, four models were compared based on Widaman’s [55] paradigm, using Mplus 8.1 with MLM maximum likelihood parameter estimation. Because the two other burnout questionnaires do not measure secondary symptoms, we only focussed on the BAT-C. Model 1 (see Figure 1). The MTMM model, or the correlated traits–correlated methods model (CT-CM), acts as the baseline model to which all other models were compared. It is also the least restrictive model because it allows both traits (i.e., the various burnout dimensions) and methods (i.e., measures such as the BAT-C, MBI-GS and OLBI) to correlate freely with each other, whilst the traits and methods are uncorrelated. The CT-CM model assumes that each item is determined by its trait factor, method factor, and an error term. Model 2, the method model, also known as the no traits–correlated method model (NT-CM), is based on the assumption that the method used—either the BAT-C, MBI-GS or OLBI—best describes the structure of the data. Each item is thus only determined by its method (i.e., the measure it stems from) and an error term. Convergent validity was evaluated by comparing Model 2 with Model 1. If Model 1 fits the data better, then there is evidence for convergent validity because this supports the assumption that independent measures of the same trait are correlated [56]. 

Models 3 and 4 are constrained versions of Model 1. In Model 3, known as the perfectly correlated traits–correlated method model (PCT-CM), the traits are perfectly correlated and hence fixed to 1, whereas in Model 4, known as the correlated traits–perfectly correlated method model (CT-PCM), the methods are perfectly correlated and hence fixed to 1. Both models served as a test of discriminant validity because it can be assumed that if the traits and measures are independent, a model that allows them to correlate freely, although not perfectly, should provide a better fit. In case Model 3 fits better to the data than Model 1, discriminant validity in terms of traits is supported, and when Model 4 fits better to the data than Model 1 discriminant validity in terms of methods is supported. In order to evaluate goodness-of-fit the same fit indices are used as mentioned above. 

*Discriminant validity with measures of work engagement, workaholism, and job boredom.* In order to evaluate the discriminant validity of the BAT vis-à-vis work engagement, workaholism, and job boredom the guidelines of Fornell and Larcker [57] were followed, using Mplus 8.1. Given two random factors, evidence of discriminant validity is obtained when the Average Variance Extracted (AVE)—the amount of variance of a construct in relation to the amount of variance due to its measurement error—of factor 1 and factor 2 exceeds the squared correlation (R^2^) between the two factors. In order to test this assumption, a general CFA-model was evaluated, using MLM maximum likelihood parameter estimation, in which each item was represented by its factor. In our case, the AVE of the BAT-C, BAT-S and the depression subscale of the 4-DSQ should be larger than their squared correlations (R^2^) with the UWES (work engagement), the EW (excessive workaholism), and CW (compulsive workaholism) subscales of DUWAS and the DUBS (job boredom), respectively.

#### 3.1.4. Results

##### Factorial Validity: Exploring the Factor Structure

For the core dimensions, measured by the BAT-C, KMO equalled 0.96 (“superb” according to Hutcheson & Sofroniou [58]) and Bartlett’s test of sphericity was significant (χ^2^ = 13833.09, df = 253, *p* < 0.001), indicating that the correlations between items contained enough common variance to make EFA useful. All 23 items loaded above 0.47 on the first unrotated factor (range: 0.47–0.85), which explained 52.13% of the common variance. Based upon the eigenvalues (using Kaiser’s criterion of 1) and the scree plot a four-factor solution was retained, explaining 67.66% of the common variance. The correlations between the factors ranged between 0.50 and 0.64. All items, except one (item 6; “*inability to be active*”), loaded above 0.40 on their respective factors (range 0.40–0.92) and *no* cross-loading items were identified. The four factors could be interpreted as the four core dimensions: Exhaustion, mental distance, and impaired emotional and cognitive control, respectively. Tellingly, the first factor (exhaustion) explains by far the most variance.

For the secondary dimensions, measured by the BAT-S and the depression subscale of the 4-DSQ, KMO equals 0.92 and Bartlett’s test of sphericity was significant as well (χ^2^ = 7610.04, df = 120, *p* < 0.001). Based upon the eigenvalues and the scree plot, a two-factor solution was retained, explaining 54.89% of the common variance. All items loaded above 0.50 on their respective factors and *no* cross-loading items were identified. The first factor (range of loadings 0.50–0.78) could be interpreted as psychological and psychosomatic complaints or distress, while the second factor (range of loadings 0.71–0.92) could be interpreted as depressed mood^3^. Both factors correlated 0.54. Since psychological and psychosomatic complaints clearly and unambiguously constituted one factor, both types of complaints were merged into one scale.

##### Factorial Validity: Confirming the Factor Structure

For the core symptoms, Model 1 did not fit the data, whilst Models 2 and 3 showed a much better and similar fit to the data. For the secondary symptoms, Model 4 had the worst fit to the data. Model 5—which included a depression factor and the combined psychological and psychosomatic complaints factor (distress)—had a much better fit, albeit not optimal since CFI (0.88), TLI (0.86) were slightly below 0.90 and RMSEA (0.09) exceeded 0.08. However, when inspecting the Modification Indices, it appeared that allowing the error terms of two overlapping items for “depressed mood” (i.e., “I wish I was dead” and “It would be better if I was dead”) to correlate would improve model fit. Given the strong overlapping content of these items, this adjusted model (Model 5a) was also tested. The model fit improved as a result of this re-specification (see Table 1). Loadings on the distress factor ranged from 0.61–0.81 and on the depression factor from 0.67–0.88, whereas both factors were correlated 0.66.

When combining both the core and secondary symptom dimensions, all models showed a good and almost identical fit to the data. However, Model 6 fitted the data significantly better than Model 7 and Model 8, and Model 8 fitted the data significantly better than Model 7. Furthermore, the pattern of the latent correlations of Model 6 provided evidence for a distinction between core and secondary symptoms of the BAT. Notably, the four core symptom dimensions were more strongly related with each other (ranging from 0.61 to 0.74) than with the two secondary core dimensions (ranging from 0.57 to 0.64). The only exception was the strong latent correlation between exhaustion and distress (i.e., 0.80). Hence, the theoretically assumed distinction between core and secondary dimensions seems psychometrically viable.

#### 3.1.5. Reliability

Overall, the internal consistencies of the BAT-C and its four subscales were well above 0.70. Cronbach’s alpha ranged from 0.90 to 0.92 for the subscales (i.e., exhaustion: 0.92, mental distance: 0.91, cognitive impairment: 0.92, and emotional impairment: 0.90) and was 0.95 for the total BAT-C. For the composite BAT-S, Cronbach’s alpha was 0.90, whereas for psychological and psychosomatic complaints values for alpha were 0.81 and 0.85, respectively.

#### 3.1.6. Construct Validity

##### Convergent and Discriminant Validity with Other Burnout Measures

As expected, Model 1 had the best overall fit (see Table 2 and Figure 1) as illustrated by the most favorable χ^2^ to degrees of freedom ratio (4.43), the highest CFI (0.91) and TLI (0.90), and the lowest RMSEA (0.05).

This supports the convergent and discriminant validity of the BAT-C vis-à-vis the MBI-GS and OLBI. Furthermore, as can be seen in Table 2, when comparing Model 2 with Model 1, the inclusion of traits in Model 1 significantly improved model fit and supported the convergent validity of the BAT-C. Furthermore, the statistically significant differences in fit between Model 3 and Model 1, and between Model 4 and Model 1, provided support for the discriminant validity of the BAT-C, both in terms of traits and methods. This means that the four dimensions of burnout are related to each other but still differ in a meaningful way indicating that, for instance, exhaustion is not equivalent to mental distance despite that both are strongly correlated. It also means that each of the three burnout instruments provided additional information about the different dimensions. This is illustrated by the latent correlations in the MTMM model (Model 1), which ranged from −0.33 to 0.88 for the traits (i.e., the dimensions) and 0.87 to 0.89 for the methods (i.e., the questionnaires). To some extent, large correlations between the methods can be expected given the content of the scales; after all, all three scales intend to measure burnout using a self-report questionnaire. In sum, our results show that while there is some convergence in the core dimensions of burnout as measured by each of the questionnaires, divergence exists as well, indicating their unique and independent contributions to the measurement of burnout. 

##### Discriminant Validity with Measures of Work Engagement, Workaholism, and Job Boredom

As can be seen in Table 3, the AVE of the BAT-C, BAT-S, and the depression subscale of the 4-DSQ were larger than their squared correlations (R^2^) with the UWES, the EW, and CW, subscales of DUWAS and the DUBS, respectively. An exception was only observed for the two scales of the DUWAS (excessive and compulsive working) and for the core and secondary distress symptoms of the BAT. 

This means that both aspects of work addiction as well as the core and secondary symptoms of the BAT cannot be distinguished from each other. This is unsurprising because in both cases the scales refer to a common underlying concept, namely work addiction and burnout, respectively.

Burnout, as assessed by the BAT, can thus successfully be discriminated from other well-being constructs such as engagement, workaholism, or job boredom, albeit that the core symptoms of burnout cannot discriminated from the secondary symptoms. This also goes in line with Table 1 that showed that the second-order Models 7 and 8 fitted the data well, indicating that both core and secondary symptoms refer to one underlying condition: Burnout. Furthermore, the direction of the correlations also revealed that—as expected—burnout is *negatively* related to engagement and *positively* related to workaholism and job boredom.

## 4. Discussion

The current paper contributes to the conceptualization and operationalization of burnout. It introduces s a novel definition of burnout and presents evidence for the validity and reliability of the Burnout Assessment Tool (BAT). Although burnout is heavily researched, with over 80,000 publications on the topic [40], its operationalization is one of the least-investigated topics in burnout research [59] and continues to be debated [60]. Most researchers use the MBI, which is considered the golden standard despite its conceptual, psychometric, and practical shortcomings. Therefore, we embarked on an attempt to develop a viable alternative. 

### 4.1. Conceptualization of Burnout 

Our first aim was to develop a more comprehensive conceptualization of burnout, adhering to the accumulating evidence that its conceptualization in terms of the MBI is flawed. Using a dialectic perspective, seven dimensions were derived from semi-structured interviews with professionals and integrated into the conceptual framework of Schaufeli and Taris [12]. As a result, four core dimensions emerged, three of which refer to the inability to invest energy (i.e., exhaustion, cognitive and emotional impairment) and one referring to the unwillingness to invest energy (i.e., mental distance). Moreover, three atypical secondary dimensions were distinguished that often co-occur with the core symptoms (i.e., depressed mood, psychological distress, and psychosomatic complaints). This clustering into core and secondary dimensions is unique in the sense that previous literature usually focused on burnout as either mere exhaustion (e.g., [18]), or as a syndrome consisting of two (e.g., [23]) or three dimensions (e.g., [1]). 

Please note that in the development of the MBI, in-depth interviews also played a role [61]. However, the MBI was developed purely inductively since it was *exclusively* based on interviews. In contrast, theoretical notions played a major role in the development of the BAT, since a combination of an inductive and a deductive approach was used. Furthermore, in the case of the MBI, basically healthy employees were interviewed about their own symptoms, whereas in the case of the BAT, professionals were interviewed about the symptoms of their patients or clients who suffered from severe burnout. Finally, the BAT is also based on an analysis of existing burnout questionnaires. 

Our results show that while exhaustion is the heart of the burnout syndrome, it is not the only core symptom. First, the anticipated importance of cognitive malfunctioning and deficits was confirmed by the expert interviews and our psychometric analyses. This is in line with Oosterholt, Maes et al., [62], who showed that after 1.5 years, burnout patients still show minor cognitive impairments, while another study by van Dam, Keijsers, Eling, and Becker [63] showed that although burnout patients show improvement in their functioning after two years, their cognitive performance is still inferior compared to healthy individuals. In addition to cognitive impairment, which seem to remain even when other burnout symptoms have disappeared, emotional impairment emerged from our expert interviews and psychometrical analyses as a novel dimension of burnout. It is not the same as emotional exhaustion, an aspect of exhaustion often seen as the core component of burnout [64]. Moreover, emotional regulation and burnout were found to be positively associated with each other, especially in emotionally demanding jobs such as in teaching or nursing [65]. Additionally, we doubted a priori if reduced professional efficacy can be considered a constituent element of the burnout syndrome. Indeed, our experts considered reduced professional efficacy as a consequence, rather than as an integral part of burnout. This is also in line with a longitudinal study by Taris, Le Blanc, Schaufeli, and Schreurs [66] who showed that, using the MBI, exhaustion leads to cynicism and cynicism, in turn, leads to lack of professional efficacy. 

With regards to the secondary dimensions, the presence of specific distress symptoms was suggested before by van der Heiden and Hoogduin [7]. The results of our study confirm their point of view; it seems that distress (i.e., psychological distress and psychosomatic complaints) constitutes an additional, secondary dimension of burnout. Furthermore, depressed mood appears to be a separate secondary dimension of burnout, independently from distress. Distress and depressed mood are considered secondary because they do not fit the conceptual framework of Schaufeli and Taris [12], albeit that practitioners see both secondary dimensions as (a non-essential) part of the syndrome. A study by Kakiashvili et al. [10] confirms this point of view. Their study on the differences between burnout and an atypical depressive disorder reveals that a depressed mood only occasionally occurs among burned out individuals, whereas it is a characteristic feature of a depressive disorder. 

### 4.2. The Development and Psychometric Evaluation of the BAT

Our second aim was to develop and test the Burnout Assessment Tool (BAT), based on this new conceptualization. The BAT consists of 33 items and measures the core dimensions (BAT-C) of burnout as well as the secondary dimensions (BAT-S). Depressed mood was not included in the BAT, because other well-validated scales adequately capture this dimension. There are also scales to measure the two other secondary dimensions. However, usually these scales are relatively long and rarely contain all symptoms which are specific to burnout. Hence, for reasons of economy, we chose to develop our own measures for psychological distress and psychosomatic complaints. 

In terms of factorial validity, three conclusions can be drawn. First, for the BAT-C, our hypothesized four-factor structure is confirmed. However, given the strong loadings on the first unrotated factor (above 0.62) and the high correlations among the four factors (ranging from 0.76 to 0.88), a second-order model, representing a syndrome, was tested, which performed equally well as the original four-factor model. However, conceptually speaking, the second-order model is preferred above the four-factor correlated model because—as noted above—burnout was conceived as a *syndrome* that by definition consists of a set of related symptoms that refer to one underlying psychological condition. Clearly, the notion of a burnout syndrome is compatible with a second-order model in which the second-order factor represents this underlying condition (i.e., burnout) and the first-order factors represent its specific symptoms. This implicates that the composite score on the BAT can be used as an indicator of burnout. In contrast, neither the MBI nor the OLBI produces a single burnout score; instead, multiple scores are obtained that are indicative for different aspects of burnout. 

Secondly, the results of our EFA show that for the BAT-S, a distinction between psychological distress and psychosomatic complaints cannot be made. This was surprising given that such a distinction has often been made before, for instance by Terluin et al. [32], who distinguished distress—related to our conceptualization of psychological distress—from somatization—related to our conceptualization of psychosomatic complaints. Moreover, the authors of the Symptom Checklist 90-Revised (SCL-90R) [67] even discriminate between nine different subtypes of distress such as sleep or somatization. However, the SCL-90R stipulates that the different types of distress are interrelated and a composite, total score can be computed, reflecting the person’s overall level of distress. Typically, the interviewed practitioners did *not* make a clear distinction between the different types of distress symptoms and the latent correlation between psychosomatic complaints and psychological distress is high (*r* = 0.81). We conclude that, although different types of distress may exist, they are highly related to each other (at least in a representative sample of the working population), and refer to a general form of distress. Hence, it seems justified to compute a composite distress score. Yet, it appears, from our CFA that distress can be discriminated from depressive symptoms, as assessed with the 4DSQ. 

Thirdly, when the comprehensive conceptualization of burnout is tested, including the core and secondary burnout symptoms, all models achieved an optimal fit. The six-factor model (i.e., exhaustion, mental distance, cognitive and emotional impairment, distress symptoms, and depressed mood) has the best fit, followed by a second-order model in which the core and secondary dimensions are discriminated from each other. Nonetheless, an examination of the latent correlations in the six-factor model shows that the core dimensions are more strongly related to each other than to the secondary dimensions, with the exception of the correlation between exhaustion and distress complaints. Tellingly, Verbraak, Kleyweg, van den Griendt, and Hoogduin [35], who examined the factorial validity of the BO-NKS, observed the same result. They distinguished two types of distress complaints—similar to our distinction between psychological distress and psychosomatic complaints —and found that both correlate higher with exhaustion than with each other. Given the adequate fit, and the results from the latent correlations in the six-factor model, the second-order model, which discriminates between the core and secondary dimensions, is preferred on theoretical grounds. Namely, (a) burnout is considered to be a *syndrome*—which is compatible with the idea of a second-order model; and (b) a distinction between core and secondary burnout symptoms is in line with our definition of burnout that is grounded in our conceptualization of burnout as well as in our qualitative analyses (Part 1). This is confirmed by a recent study of the BAT in a representative sample of Japanese workers [68]. This study corroborated that a second-order factor model including only the core symptoms as well as a second-order factor model including the core symptoms plus the secondary symptoms fitted well to the data. Moreover, another recent study found that the former second-order factor model was invariant across seven cross-national representative samples from Austria, Belgium, Finland, Germany, Ireland, Japan, and The Netherlands [69]. Finally, using Rasch analysis it was shown that the core symptom-dimensions of the BAT constitute a unidimensional scale [70]. This means that also from the rather rigorous Rasch perspective, a single composite score of the BAT can be computed, which is indicative for a person’s level of burnout; the higher the score, the higher the level of burnout. This study also showed that the total BAT-score works invariantly for women and men, for younger and older respondents, and for respondents from different countries.

The internal consistency of the BAT in terms of Cronbach’s alpha is in the current study higher than 0.90, which is considered very good [71], and slightly better than the coefficients for the MBI (0.84 to 0.92) and the OLBI (0.78 and 0.85). 

Three conclusions can be drawn regarding the construct validity of the BAT. First, the MTMM-framework provides evidence that the BAT-C measures burnout, and thus converges with the MBI and OLBI. This convergence in terms of method (i.e., questionnaire) is not surprising, with latent correlations ranging from 0.87 to 0.89, given the content of the questionnaires. In essence, all three measurements seek to assess burnout in an organizational context using self-report items that are scored on a Likert scale. The latent correlations between the various burnout dimensions are also moderate to high (0.49 to 0.87), which provides evidence that, indeed, burnout can be considered as a syndrome that consists of multiple, interrelated dimensions. The only exception is professional efficacy. Again, this scale seems to be at odds, with latent correlations with the other burnout dimensions of around −0.33 to −0.15. Additionally, the MTMM-framework also indicates that there is some divergence in the way in which each questionnaire conceptualizes burnout, which is not surprising given their different conceptualizations. Additionally, the recent Japanese study of Sakakibara et al. [68] who also used the MTMM framework corroborated the current findings by demonstrating convergent and discriminant validity of the BAT vis-à-vis the MBI-GS. Taken together, these results concur with the conclusions drawn by Halbesleben and Demerouti [72] who used a similar framework as well to test the convergent and discriminant validity of the OLBI and MBI-GS. 

Secondly, the results further suggest that the construct of burnout, as measured by the BAT, can be discriminated from other well-being constructs, such as engagement (i.e., UWES), job boredom (i.e., DUBS), or workaholism (i.e., DUWAS). Using the same questionaries and methodology, a recent Japanese study corroborated the discriminant validity of the BAT vis-à-vis work engagement and workaholism [68]. These results concur with Schaufeli, Taris, and Van Rhenen’s [73], who conclude that engagement, workaholism, and burnout can be distinguished from each other empirically, as well as with Schaufeli and Salanova [45] who theorize about the differences between burnout, job boredom, and engagement. 

Third, as Table 3 indicates, depressed mood, as assessed with a subscale of the 4DSQ, can be distinguished from the core symptoms of burnout as well as from secondary psychological distress. This result seems at odds with the idea that burnout and depression completely overlap and that burnout is, in fact, an atypical depression [8]. Based on our findings, we recommend using the depression subscale of the 4DSQ in addition to the BAT.

### 4.3. Limitations and Suggestions for Future Research 

As in every study, some limitations need to be addressed. Theoretically, the renewed conceptualization is based on the expertise of those who deal professionally with burnout patients and not on those who suffer from it. While a practitioner’s point of view can be valuable because it is more objective and comprehensive, future research should focus on validating their point of view with the experiences and symptoms of those who suffer from burnout. Furthermore, our definition of burnout may implicate an evolution from exhaustion to cognitive and emotional impairment to mental distance, and back. Given our cross-sectional design, this could not be tested. Future longitudinal research should focus on the way the burnout syndrome unfolds over time. Empirically, our study only looked into factorial validity, reliability, and construct validity. Given the novelty of our study, this was a first step in validating the BAT. Although the results are promising and seem to be corroborated by three very recent studies [68,69,70], they are still preliminary. Additional, more elaborate testing should take place in the future in order to evaluate the validity of the BAT. For instance, future research could focus on how useful it is to discriminate between the different subscales, because they could potentially correlate differently with other constructs. More specifically, the relationships between the BAT and other constructs (such as job demands, job resources, personality or work-related outcomes such as performance) needs to be examined. In addition, convergent validity of the secondary symptoms should be more closely examined in future studies, for instance, by using other well-validated distress scales such as the General Health Questionnaire [74]. Last, but not least, the BAT was tested in a Flemish cultural context—it remains to be investigated how it behaves in other cultural settings, albeit that the first, preliminary cross-national results seem promising [69]. 

Practically, two drawbacks are identified. First, as noted before, a single burnout score can be derived from the BAT, which basically allows making a distinction between healthy employees and those who run a large risk of burning-out. However, such a distinction requires clinically validated cut-off scores, which are not yet available for the BAT. Using Relative Operating Characteristics Analysis, or ROC analysis [75], an optimum cut-off value for the BAT can be calculated to discriminate “cases” from “non-cases”, taking into account both its specificity (the probability of a negative result) as well as its sensitivity (the probability of a positive result). Such cut-off points are important for evaluating the effectiveness of interventions for burnout, for screening employees who are at risk for burnout in order to target them with preventive measures, and finally for determining the prevalence of burnout [76]. Moreover, it would also allow focusing on how burnout patients respond differently than other, for instance depressed, patients, or healthy workers. The BAT’s practical usability, especially as a preventive screening tool and diagnostic assessment instrument, will be promoted when future research would establish clinically validated cut-off points, by using patient samples. It should be emphasized though that a comprehensive burnout diagnoses cannot be made exclusively on the basis of the BAT or any other questionnaire, for that matter. This requires a thorough clinical interview by a trained professional, yet the BAT can be useful as an additional source of information. Second, especially for practical use of the BAT, predictive validity is paramount, all the more since the MBI is, for instance, unable to predict future long-term sickness absence [77].

## 5. Conclusions

The results of our study provide initial evidence for a new conceptualization of burnout and an associated measure, the Burnout Assessment Tool. Specifically, evidence is found for the reliability and factorial and construct validity of the BAT. By tackling two essential flaws in the MBI (i.e., conceptualization and psychometric shortcomings), and providing a starting point for overcoming the third flaw (i.e., practical applicability) through using a single, composite burnout score, a boost can be given not only to burnout research, but also to the assessment of burnout in practice. Accordingly, our results suggest that the BAT can be seen as a viable, alternative burnout measure, that assesses the burnout syndrome as such (total score), as well as its core components and secondary symptoms. Ultimately, by building on the proposed reconceptualization of burnout, the BAT may contribute to a better understanding of the phenomenon.

## Figures and Tables

**Table 1:** Model fit indices for the different BAT models.

Model		χ^2^	S-Bχ^2^	df	CFI	TLI	RMSEA [90% CI]		Δχ^2^	*p*
*Core symptoms*										
1	Unidimensional model	2960.13	1.29	230	0.73	0.70	0.13 [0.12–0.13]			
2	Correlated 4-factor model	773.54	1.28	224	0.95	0.94	0.06 [0.05–0.06]	2 vs. 1	1700.46	<0.0001
3	Second-order model (4 first order, 1 second order)	776.79	1.28	226	0.95	0.94	0.06 [0.05–0.06]	3 vs. 1 3 vs. 2	1522.52 3.25	<0.0001 0.20
*Secondary symptoms*										
4	Unidimensional model	1963.62	1.29	104	0.70	0.65	0.15 [0.15–0.16]			
5	Correlated 2-factor model	852.11	1.30	103	0.88	0.86	0.10 [0.09–0.10]	5 vs. 4	5482.03	<0.0001
5a	Adjusted correlated 2-factor model	500.48	1.29	102	0.94	0.92	0.07 [0.07–0.08]	5a vs. 4 5a vs. 5	2413.39351.63	<0.0001
*Core & secondary symptoms*										
6	Correlated 6-factor model	2244.29	1.25	687	0.91	0.90	0.06 [0.05–0.06]			
7	Second-order model (6 first order, 1 second-order)	2309.48	1.25	696	0.91	0.90	0.06 [0.05–0.06]	7 vs. 6	65.19	<0.0001
8	Second-order model (6 first order, 2 second-order)	2293.77	1.25	695	0.91	0.90	0.06 [0.05–0.06]	8 vs. 6 8 vs. 7	49.4815.71	<0.0001 <0.0001

Note. χ^2^ = chi-square; S-Bχ^2^ = Satorra–Bentler scaling factor for chi-square; df = degrees of freedom; CFI = comparative fit index; TLI = Tucker–Lewis index; RMSEA = root mean square error of approximation; Δχ^2^ = difference in chi-square; Δdf = difference in degrees of freedom; *p* = *p*-value. The factor-loading matrices of the models and all correlations between the latent variables are available upon request from the first author.

**Table 2:** Model fit indices for the Multi-Trait, Multi-Method framework of the BAT.

Model		χ^2^	df	S-Bχ^2^	CFI	TLI	RMSEA [90% CI]		Δχ^2^	Δdf	*p*
1	CT-CM model	5803.79	1310	1.1963	0.91	0.90	0.05 [0.04–0.05]				
2	NT-CM model	10020.64	1367	1.1974	0.83	0.82	0.07 [0.06–0.07]	2 vs. 1	4134.88	57	<0.0001
3	PCT-CM model	7022.20	1313	1.1973	0.89	0.88	0.05 [0.04–0.05]	3 vs. 1	896.35	3	<0.0001
4	CT-PCM model	11203.54	1320	1.1973	0.80	0.79	0.07 [0.06–0.07]	4 vs. 1	4871.58	10	<0.0001

Note. χ^2^ = chi-square; S-Bχ^2^ = Satorra–Bentler scaling factor for chi-square; df = degrees of freedom; CFI = comparative fit index; TLI = Tucker–Lewis index; RMSEA = root mean square error of approximation; Δχ^2^ = difference in chi-square; Δdf = difference in degrees of freedom; *p* = *p*-value.

**Table 3:** Average Variance Extracted (AVE) scores and squared correlations for different measures.

		AVE				R^2^			
			1	2	3	4	5	6	7
1	Engagement (UWES)	0.76	-						
2	Workaholism: Excessive working (DUWAS)	0.51	0.07	-					
3	Workaholism: Compulsive working (DUWAS)	0.53	0.00	0.59	-				
4	Job boredom (DUBS)	0.58	0.41	0.12	0.05	-			
5	Core symptoms of burnout (BAT-C)	0.51	0.42	0.03	0.15	0.21	-		
6	Secondary burnout symptoms: Psychological distress (BAT-S)	0.51	0.17	0.07	0.23	0.03	0.62	-	
7	Secondary burnout symptoms: Depressed mood (4DSQ)	0.52	0.15	0.02	0.12	0.10	0.38	0.39	-

Note. AVE = Average Variance Extracted, R^2^ = squared correlations.

## Appendix A

**Table A1:** Burnout questionnaires.

Questionnaire	Subscale	# Items
*Validated*		
Bergen Burnout Inventory (BBI; Salmela-Aro, Rantanen, Hyvönen, Tilleman, & Feldt, 2011)	Exhaustion	5
	Cynicism	5
	Inadequacy	5
Burnout Measure (BM; Pines & Aronson, 1981)	Exhaustion	21
BurnOut-Neuratshenia Complaints Scale (BONKS; Verbraak, van de Griendt & Hoogduin, 2006)	Mental fatigue	3
	Physical fatigue	3
	Mental fatigability	3
	Physical fatigability	4
	Muscle pain	5
	Dizziness	4
	Tension headaches	6
	Poor sleep	5
	Inability to relax	4
	Irritability	5
	Gastro-intestinal symptoms	4
Copenhagen Burnout Inventory (CBI; Kristensen, Borritz, Villadsen, & Christensen, 2005)	Work-related burnout	5
Spanish Burnout Inventory (SBI; Gil-Monte & Faúndez, 2011)	Work enthusiasm	5
	Psychological exhaustion	4
	Indolence	6
	Guilt	4
Granada Burnout Questionnaire (GBQ; De la Fuente, et al., 2013)	Emotional exhaustion	8
	Depersonalization	7
	Personal accomplishment	11
Maslach Burnout Inventory-General Survey (MBI-GS; Schaufeli, Leiter, Maslach & Jackson, 1996)	Exhaustion	5
	Cynicism	5
	Professional efficacy	6
Oldenburg Burnout Inventory (OLBI; Demerouti, Bakker, Vardakou & Kantas, 2003)	Exhaustion	8
	Disengagement	8
Shirom Melamed Burnout Measure (SMBM; Shirom & Melamed, 2006)	Emotional exhaustion	4
	Chronic fatigue	4
	Cognitive weariness	6
4-Dimensional Questionnaire (4-DSQ; Terluin, van Marwijk et al., 2006)	Distress	16
	Depression	6
	Anxiety	12
	Somatization	16
*Non-validated*		
Boudreau Burnout Questionnaire (BBQ; Boudreau, Cahoon & Wedel, 2006)	Emotional exhaustion	10
	Depersonalization	10
	Lack of personal accomplishment	10
	Fatality	10
Instrument for the early detection of burnout (FOD, 2017)	Physical symptoms	4
	Cognitive-affective symptoms	12
	Behavioral symptoms	5
Hamburg Burnout Inventory (HBI; Burisch, 2017)	Emotional exhaustion	5
	Distance	4
	Personal accomplishment	3
	Depressive reaction	3
	Helplessness	4
	Inner void	4
	Tedium	5
	Inability to unwind	3
	Overtaxing oneself	5
	Aggressive reaction	3

## Appendix B

The following statements are related to your work situation and how you experience this situation. Please state how often each statement applies to you.

**Table A2:** Core Symptoms (BAT-C).

	Never	Rarely	Sometimes	Often	Always
*Exhaustion*					
1. At work, I feel mentally exhausted	☐	☐	☐	☐	☐
2. Everything I do at work requires a great deal of effort	☐	☐	☐	☐	☐
3. After a day at work, I find it hard to recover my energy	☐	☐	☐	☐	☐
4. At work, I feel physically exhausted	☐	☐	☐	☐	☐
5. When I get up in the morning, I lack the energy to start a new day at work	☐	☐	☐	☐	☐
6. I want to be active at work, but somehow, I am unable to manage	☐	☐	☐	☐	☐
7. When I exert myself at work, I quickly get tired	☐	☐	☐	☐	☐
8. At the end of my working day, I feel mentally exhausted and drained	☐	☐	☐	☐	☐
*Mental distance*					
9. I struggle to find any enthusiasm for my work	☐	☐	☐	☐	☐
10. At work, I do not think much about what I am doing and I function on autopilot	☐	☐	☐	☐	☐
11. I feel a strong aversion towards my job	☐	☐	☐	☐	☐
12. I feel indifferent about my job	☐	☐	☐	☐	☐
13. I’m cynical about what my work means to others	☐	☐	☐	☐	☐
*Cognitive impairment*					
14. At work, I have trouble staying focused	☐	☐	☐	☐	☐
15. At work I struggle to think clearly	☐	☐	☐	☐	☐
16. I’m forgetful and distracted at work	☐	☐	☐	☐	☐
17. When I’m working, I have trouble concentrating	☐	☐	☐	☐	☐
18. I make mistakes in my work because I have my mind on other things	☐	☐	☐	☐	☐
*Emotional impairment*					
19. At work, I feel unable to control my emotions	☐	☐	☐	☐	☐
20. I do not recognize myself in the way I react emotionally at work	☐	☐	☐	☐	☐
21. During my work I become irritable when things don’t go my way	☐	☐	☐	☐	☐
22. I get upset or sad at work without knowing why	☐	☐	☐	☐	☐
23. At work I may overreact unintentionally	☐	☐	☐	☐	☐

**Table A3:** Secondary Symptoms (BAT-S).

	Never	Rarely	Sometimes	Often	Always
*Psychological complaints*					
1. I have trouble falling or staying asleep	☐	☐	☐	☐	☐
2. I tend to worry	☐	☐	☐	☐	☐
3. I feel tense and stressed	☐	☐	☐	☐	☐
4. I feel anxious and/or suffer from panic attacks	☐	☐	☐	☐	☐
5. Noise and crowds disturb me	☐	☐	☐	☐	☐
*Psychosomatic complaints*					
6. I suffer from palpitations or chest pain	☐	☐	☐	☐	☐
7. I suffer from stomach and/or intestinal complaints	☐	☐	☐	☐	☐
8. I suffer from headaches	☐	☐	☐	☐	☐
9. I suffer from muscle pain, for example in the neck, shoulder or back	☐	☐	☐	☐	☐
10. I often get sick	☐	☐	☐	☐	☐
